# LRRK2 deficiency induced mitochondrial Ca^2+^ efflux inhibition can be rescued by Na^+^/Ca^2+^/Li^+^ exchanger upregulation

**DOI:** 10.1038/s41419-019-1469-5

**Published:** 2019-03-19

**Authors:** Marthe H. R. Ludtmann, Marko Kostic, Amy Horne, Sonia Gandhi, Israel Sekler, Andrey Y. Abramov

**Affiliations:** 1 Royal Veterinary College, 4 Royal College St, Kings Cross, London, NW1 0TU UK; 2 Department of Clinical and Movement Neuroscience, UCL Institute of Neurology, London, WC1N 3BG UK; 3 Department of Physiology and Cell Biology, Faculty of Health Sciences, Ben-Gurion University of the Negev, Beer-Sheva, 84105 Israel; 4 The Francis Crick Institute, 1 Midland Road, King’s Cross, London, NW1 1AT UK

## Abstract

Variants of leucine-rich repeat kinase 2 (*lrrk2*) are associated with an increased risk in developing Parkinson’s disease (PD). Mitochondrial dysfunction and specifically mitochondrial Ca^2+^ handling has been linked to the pathogenesis of PD. Here we describe for the second time a mitochondrial Ca^2+^ efflux deficiency in a model displaying alterations in a PD-associated risk protein. LRRK2 deletion, inhibition and mutations led to an impaired mitochondrial Ca^2+^ extrusion via Na^+^/Ca^2+^/Li^+^ exchanger (NCLX) which in turn lowered mitochondrial permeability transition pore (PTP) opening threshold and increased cell death. The mitochondrial membrane potential was found not to be the underlying cause for the Ca^2+^ extrusion deficiency. NCLX activity was rescued by a direct (phosphomimetic NCLX mutant) and indirect (protein kinase A) activation which in turn elevated the PTP opening threshold. Therefore, at least two PD-associated risk protein pathways appear to converge on NCLX controlling mitochondrial Ca^2+^ extrusion and therefore mitochondrial health. Since mitochondrial Ca^2+^ overload has been described in many neurological disorders this study warrants further studies into NCLX as a potential therapeutic target.

## Introduction

Parkinson’s disease is a common, disabling, incurable neurodegenerative condition affecting 1% of the population over the age of 60. The past 10 years have seen a shift in the aetiological understanding of PD, moving from a nearly exclusively environmentally mediated disease towards a complex disorder with important genetic contributions^1^. One of the most extensively studied genes containing these mutations is the leucine-rich repeat kinase 2. In particular, the missense mutation G2019S has been reported to be the underlying cause for PD in 1–2% in the UK/USA rising to 10% in Ashkenazi Jews and 40% in North African Arab Berbers^2,3^. LRRK2 possesses a kinase and a ROC-GTPase domain whose exact physiological function is yet to be fully unravelled but studies provided evidence for a role of LRRK2 in vesicle trafficking, inflammatory responses, autophagy and neurite outgrowth^4–7^; with few phosphorylation substrates identified^8,9^. The multitude of pathways regulated by LRRK2 demonstrate the complexity of LRRK2 mutations and their potential effects on cellular signalling pathways. In PD, it has long been known that LRRK2 mutations (in particular G2019S) lead to an increased kinase activity and this upregulation is currently being targeted by LRRK2 kinase inhibitors as a therapeutic agent in PD^10–12^.

Mitochondrial dysfunction is strongly implicated in PD as toxic models of PD which employ mitochondrial toxins as well as the majority of proteins associated with familial forms of PD affect mitochondrial function. Recently, we have shown how another PD-associated protein, PINK1, is involved in mitochondrial Ca^2+^ homoeostasis suggesting a potential role for the recently described sodium Ca^2+^ exchanger (NCLX) in PD pathology^13–15^. While mitochondrial Ca^2+^ shuttling couples between Ca^2+^ signalling and metabolic activity, its impairment can lead to mitochondrial Ca^2+^ overload that can be a trigger for neuronal cell death. We have shown that the mitochondrial Ca^2+^ overload phenotype in PINK1 deficient cells can be rescued by a PKA/NCLX-mediated pathway^16^. Furthermore, α-synuclein has been linked to Ca^2+^-induced cell death^17,18^ suggesting that inhibition of mitochondrial Ca^2+^ efflux may be a common feature in the mechanism of neurodegeneration observed in PD. To test this hypothesis, we used LRRK2 deficient cells and assessed mitochondrial Ca^2+^ homoeostasis.

This current study established that mitochondrial Ca^2+^ homoeostasis is a common phenotype in PD and not specific to PINK1 deficiency only.

## Methods

### Animals

Wild-type and LRRK2 KO mice were obtained from breeding colonies generated by Jackson Laboratories and Sprague Dawley rat pups (1–3 days postpartum) were acquired from the University College London breeding colony. Wild-type rat of either sex were used for neuronal co-cultures and experimental procedures were performed in full compliance with the United Kingdom Animal (Scientific Procedures) Act of 1986.

### Cell culture

Mixed cultures of cortical and midbrain neurons and glial cells were prepared from postnatal pups (Control/LRRK2 KO mice or rats; day 0–3; UCL breeding colony). Midbrain were removed and placed into ice-cold Ca^2+^/Mg^2+^ free PBS (Invitrogen, UK). The tissue was minced and trypsinised (0.25%; 5 min at 37 °C), triturated and plated on poly-D-lysine-coated coverslips. The tissue was cultured in Neurobasal A medium (Invitrogen, UK) supplemented with B-27 (Invitrogen, Paisley, UK) and 2 mM GlutaMAX (Invitrogen, UK)^19^. Cultures were maintained at 37 °C in a humidified atmosphere of 5% CO_2_ and 95% air; media was changed twice weekly and maintained for a minimum of 12 days before experimental use to ensure expression of glutamatergic and other receptors. Only neurons were used for analysis. Neurons were distinguishable from glia: they appeared phase bright, had smooth rounded somata and distinct processes, and lay just above the focal plane of the glial layer. Cells were used at 12–15 days in vitro. Experimental procedures were performed in full compliance with the United Kingdom Animal (Scientific Procedures) Act of 1986.

Fibroblasts were generated from a skin punch biopsy taken under local anaesthetic following local ethical approval and full informed consent^20^. Biopsies were dissected into ~1mm pieces and cultured in DMEM, 10% FBS, 1% GlutaMAX until fibroblasts were seen to grow out from the explants. When fibroblasts reached confluency, they were detached from culture dishes using TrypleE (Invitrogen) and transferred to larger culture vessels for further expansion and cryopreservation.

### Plasmids and transfection

Preparation of human WT NCLX plasmids and site-directed mutagenesis was carried out as previously described^16,21^. Plasmids were transfected using Effectene (Qiagen) according to manufacturers protocol. NCLX plasmids were co-transfected with an empty GFP vector (pcDNA3 backbone) to allow for selection of transfected cells for experimentation and analysis.

### Live-cell imaging

For cytosolic Ca^2+^ imaging, cells were loaded for 30 min with 0.005% Pluronic and the Ca^2+^ indicator Fluo-4 AM (*K*_d_ ~ 350 nM; 5 µM). Mitochondrial Ca^2+^ was visualised by pre-loading cells with 0.005% Pluronic and either Rhod5N AM (*K*_d_ ~ 19 µM; 5 µM) or Rhod-2 AM (*K*_d_ ~ 700 nM; 5 µM) for 20 min. Whole-cell imaging was carried out in a HEPES-buffered salt solution (HBSS). Cells were loaded for 30 min with 0.005% Pluronic and CoroNa green AM (*K*_d_ ~ 80 mM; 5 µM) to visualise Na^+^.

For TMRM experiments, cells were placed in a HBSS containing 25 nM TMRM for 40 min at room temperature. TMRM was excited using the 560 nm laser line and fluorescence was measured > 580 nm using a Zeiss 710 VIS CLSM equipped with a META detection system and a ×40 oil-immersion objective. Z-stack images were obtained by confocal microscopy and the basal Δψm was measured using Zen software (Zeiss).

Mitochondrial Ca^2+^ efflux was assessed in permeabilized cells. To permeabilize, a buffer (0.137 M NaCl, 5 mM KCl, 0.7 mM NaH_2_PO_4_, 25 mM Tris-HCl, pH 7.1) containing low concentration of digitonin (20 μM) was added to the cells. Upon permeabilization, the buffer and digitonin were replaced by fresh buffer containing mitochondrial substrates (5 mM glutamate and 5 mM malate). Cells were allowed to rest for at least 5 min before measurements were taken. 20 μM CaCl_2_ was applied to assess mitochondrial Ca^2+^ handling. Confocal images were obtained using a Zeiss 710 equipped with a META detection system and a ×40 oil-immersion objective. Mitochondrial Ca^2+^ measurements were undertaken using the 543 nm laser line and 560 nm longpass filter. A 488 nm Argon laser line was used to excite Fluo-4 which was measured between 510 and 550 nm. The pinhole set to give an optical slice of ~ 2 μm and illumination intensity was kept to a minimum (at 0.1–0.2% of laser output) to avoid phototoxicity.

PTP opening threshold was determined with increasing concentrations of the electrogenic Ca^2+^ ionophore ferutinin in stepwise fashion^22^. Briefly, ferutinin induces a rise in mitochondrial calcium and the concentration of ferutinin required to induce PTP opening (confirmed by the rapid loss of TMRM signal) was determined.

### Cell toxicity experiment

Cells were incubated with propidium iodide (PI; 20 μM) and Hoechst 33342 (4.5 µM; Molecular Probes, Eugene, OR). Viable cells exclude the red fluorescent PI whereas Hoechst stains chromatin blue in all cells thus allowing dead cells to be quantified.

### Statistical analysis

Statistical analysis and exponential curve fitting were performed using Origin 9 software. Experimental data are shown as means ± SEM. Statistical analysis between samples was performed using a one-way ANOVA with Bonferroni correction. Differences were considered to be significantly different if *p* *<* 0.05.

## Results

### Ca^2+^ dysregulation in LRRK2 KO primary cultures

**Fig. 1:**
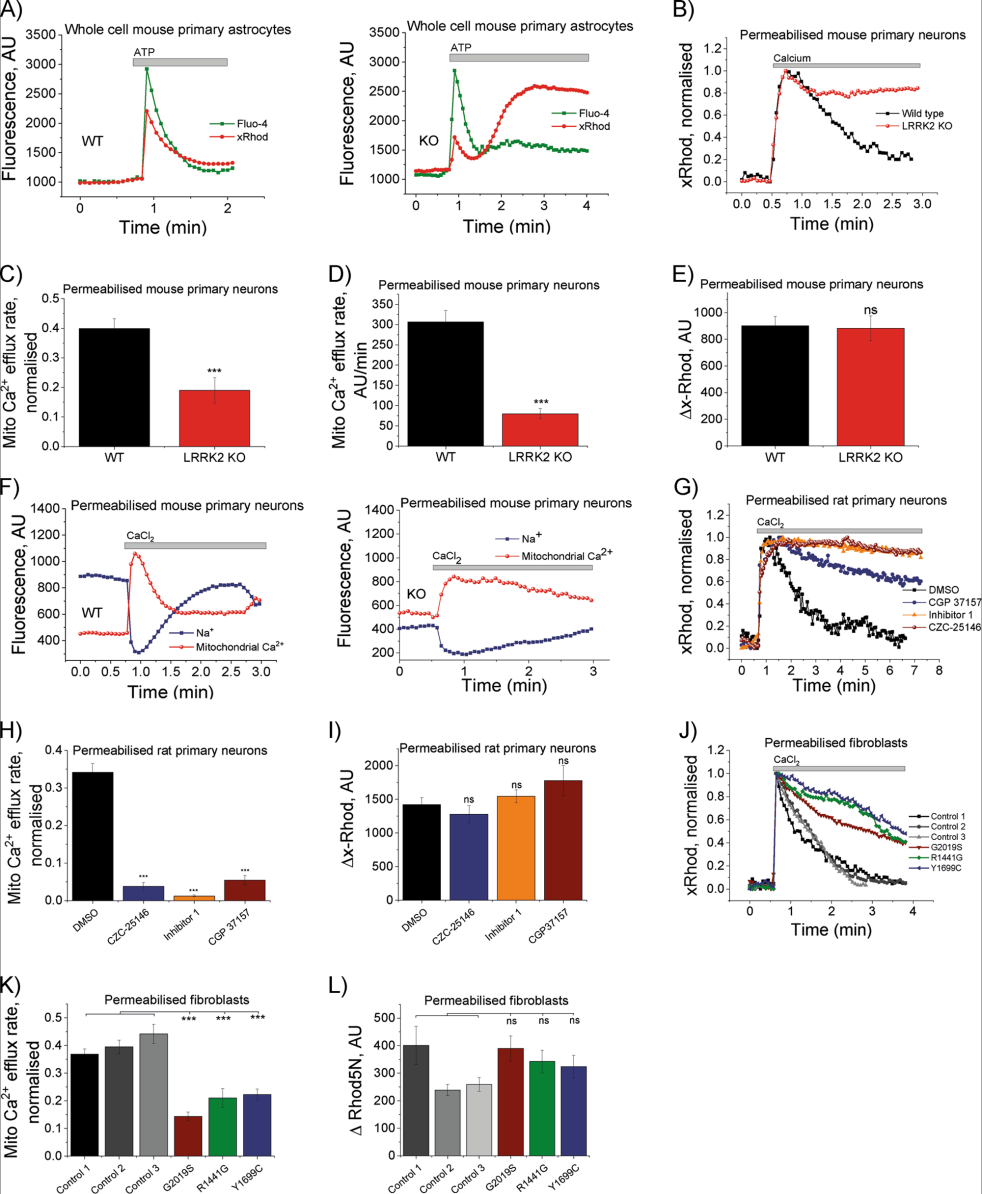
Altered Ca^2+^ homoeostasis in LRRK2 deficient cells. **a** Representative traces of either whole cell WT or LRRK2 KO primary astrocytes stimulated with ATP. **b** Representative traces of permeabilized, CaCl_2_ stimulated WT and LRRK2 KO neurons. **c** Quantification of mitochondrial Ca^2+^ efflux in WT and LRRK2 neurons (*N* = 3 experiments; *n* ≥ 45 cells). **d** Non-normalised mitochondrial Ca^2+^ efflux data mirror normalised mitochondrial Ca^2+^ efflux data (**c**). **e** Quantification of maximum mitochondrial Ca^2+^ influx in permeabilised WT and KO neurons (*N* = 3 experiments; *n* ≥ 45 cells). **f** Representative traces of sodium (CoroNA) and Ca^2+^ (xRhod1) in permeabilized, Ca^2+^-stimulated WT or LRRK2 KO neurons visualising Na^+^ and Ca^2+^ exchange. **g** Representative traces of permeabilized, CaCl_2_ stimulated WT rat neurons pre-treated with vehicle control, LRRK2-IN-1, CZC-25146 or NCLX inhibitor (CGP 37157). **h** Quantification of mitochondrial Ca^2+^ efflux in DMSO, CZC-25146, LRRK2-IN-1 or CGP37157 treated WT rat neurons (*N* = 3 experiments; *n* ≥ 39 cells). **i** Quantification of maximum mitochondrial Ca^2+^ uptake in permeabilised rat primary neurons treated with DMSO, LRRK2 or NCLX inhibitors (*N* = 3 experiments; *n* ≥ 39 cells). **j** Representative traces of permeabilized, CaCl_2_ stimulated human control and G2019S, R1441G or Y1699C fibroblasts. **k** Quantification of mitochondrial Ca^2+^ efflux in human control and LRRK2 mutation bearing fibroblasts. **l** Quantification of maximum mitochondrial Ca^2+^ uptake in permeabilised fibroblasts (*N* = 3 experiments; *n* ≥ 42 cells). ***p *<* 0.001

First we assessed whether deletion of LRRK2 leads to a dysregulation in Ca^2+^ homeostasis. To do so, we used primary neuron/glia co-cultures from the cerebral cortex/midbrain of wild-type (WT) and LRRK2 knockout (KO) mice. Both cytosolic and mitochondrial Ca^2+^ was assessed upon adenosine triphosphate (ATP) application. Intracellular Ca^2+^ levels are tightly controlled by the ER which is the main Ca^2+^ store and mitochondria which “fine-tunes” Ca^2+^ transients. Exogenous applied ATP triggers an intracellular Ca^2+^ release from the endoplasmic reticulum (ER) via P2Y receptors followed by activation of the phospholipase C and IP_3_ pathway in astrocytes^23^. The Ca^2+^ release from the ER into the cytosol leads to a simultaneous increase in mitochondrial Ca^2+^. Application of ATP led to increased cytosolic and mitochondrial Ca^2+^ in WT astrocytes and Ca^2+^ levels recovered back to basal levels typically within 1 min (Fig. 1a). However, it was noted that after a short recovery mitochondrial Ca^2+^ levels in KO astrocytes induced a delayed calcium deregulation in form of a secondary Ca^2+^ increase. The underlying reason could be induced by opening of store-operated calcium channels or energy deprivation followed by mitochondrial Ca^2+^ uptake^24^.

### Altered mitochondrial Ca^2+^ efflux in LRRK2 deficient cells

Next, we investigated the underlying reason for the observed inhibited mitochondrial Ca^2+^ efflux. Midbrain WT and KO neurons were permeabilized and mitochondrial Ca^2+^ buffering was assessed by application of exogenous CaCl_2_. Ca^2+^ was taken up by the mitochondria and the rate of mitochondrial Ca^2+^ efflux was significantly lower (0.19 ± 0.04; *p* < 0.001) in KO mitochondria when compared with WT (0.4 ± 0.03; Fig. 1b, c). The same effect was observed in cortical cells. Figure 1d exemplifies that normalised mitochondrial Ca^2+^ mirror non-normalised data. The maximal mitochondrial Ca^2+^ uptake was not significantly different in KO neurons when compared with WT neurons indicating that the slower Ca^2+^ efflux is not caused by an increased Ca^2+^ uptake (Fig. 1e).

In excitable cells, mitochondrial Ca^2+^ is predominantly removed by the sodium/Ca^2+^/lithium exchanger (NCLX) and, to a lesser extent, by other efflux pathways. NCLX translocates Na^+^ and Ca^2+^ ions across the inner mitochondrial membrane which can be visualised by loading cells with CoroNA (Na^2+^ indicator) as well as xRhod1 (mitochondrial Ca^2+^ indicator). In permeabilized WT neurons, application of CaCl_2_ resulted in an increase in mitochondrial Ca^2+^ and decrease in mitochondrial Na^+^, before Ca^2+^ is extruded and Na^+^ is taken up by the mitochondria (Fig. 1f). However, in KO neurons, NCLX-mediated mitochondrial Ca^2+^ extrusion is impaired suggesting an altered NCLX activity leading to the inhibited mitochondrial Ca^2+^ efflux.

Inhibition of kinase activity (loss of function) was employed to confirm the mitochondrial Ca^2+^ efflux deficiency is a LRRK2 mediated impairment. Therefore, two LRRK2 kinase inhibitors were employed to assess whether mitochondrial Ca^2+^ is a direct consequence of LRRK2 deficiency. To do so, WT rat primary neuron/glia co-cultures were exposed to either LRRK2 inhibitor 1 (LRRK-IN-1; 1 µM), CZC-25146 (5 µM) or DMSO vehicle control and mitochondrial Ca^2+^ homoeostasis was assessed in neurons. Inhibition of LRRK2 kinase mirrored the significant inhibited mitochondrial Ca^2+^ efflux observed in KO mitochondria (Fig. 1g, h; *p* *<* 0.001). As a positive control, the NCLX inhibitor CGP 37157 (10 µM) was employed which mirrored the significantly inhibited mitochondrial Ca^2+^ efflux similar to that observed in LRRK2 deficient models (Fig. 1g, h; 0.05 ± 0.01; *p* *<* 0.001). Figure 1i shows that maximal mitochondrial Ca^2+^ uptake was not significantly different when compared with control. Further, non-normalised data mirror the normalised efflux data.

Fibroblast from PD patients bearing LRRK2 mutations were employed to investigate whether this mitochondrial phenotype can also be observed in LRRK2-associated PD, mirroring the PINK1 phenotype. Fibroblast from patients bearing either LRRK2 G2019S (kinase domain), R1441G (ROC domain) or Y1699C (COR domain) mutation and unaffected controls were employed to assess whether mitochondrial Ca^2+^ buffering was a common phenotype in PD as well as LRRK2 deficiencies. Indeed, the mitochondrial Ca^2+^ efflux was significantly inhibited in G2019S (Fig. 1j, k; 0.14 ± 0.015; *p* *<* 0.001), R1441G (0.21 ± 0.03; *p* *<* 0.001; *n* = 3 experiments) and Y1699C (0.22 ± 0.02; *p* < 0.001) when compared with the three matched control cell lines (0.36 ± 0.02; 0.39 ± 0.02; 0.44 ± 0.03; Fig. 1j, k). Maximal mitochondrial Ca^2+^ uptake was not significantly different between cell types (Fig. 1l)

### PD-associated mutations result in lower mitochondrial Ca^2+^ handling capacity

**Fig. 2:**
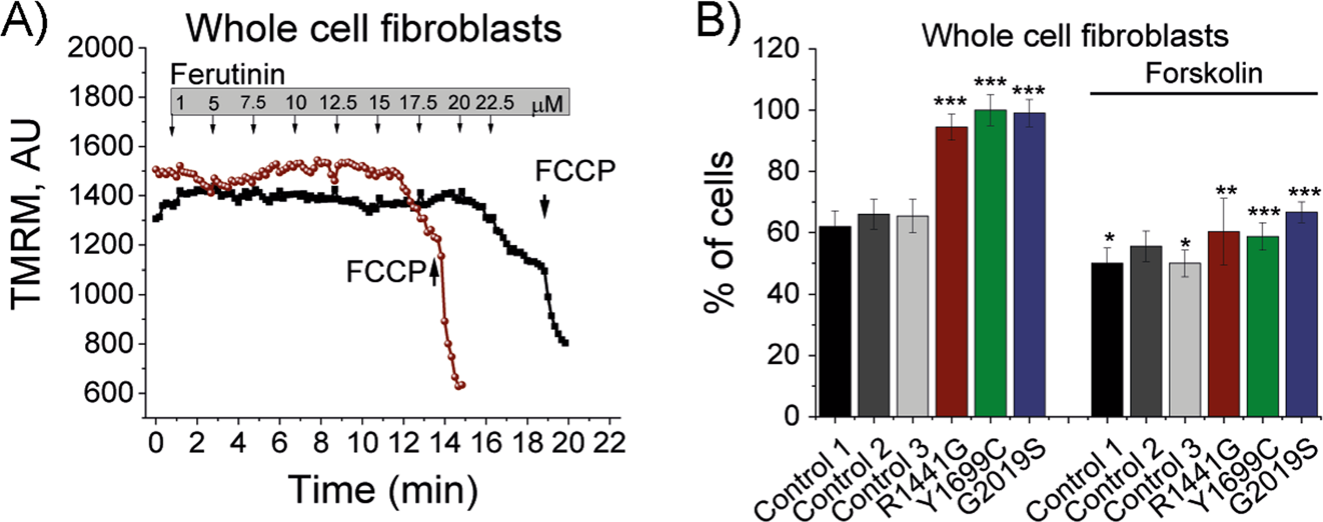
PD-associated mutations lower mitochondrial Ca^2+^ handling capacity. **a** Representative TMRM traces of control and mutant fibroblast in response to ferutinin. Ferutinin was added in a stepwise fashion at concentrations indicated in the graph. Upon full PTP opening, a steep drop in TMRM fluorescence was observed. The mitochondrial uncoupler FCCP was added as a positive control for mitochondrial uncoupling. **b** Quantification of PTP opening in control and mutant fibroblast in response to 20 μM ferutinin with and without forskolin (*N* = 4 experiments; *n* ≥ 59 cells). *p *<* 0.05; **p *<* 0.01; ***p < 0.001

The observed altered mitochondrial Ca^2+^ efflux deficiency is likely to lower the mitochondrial Ca^2+^ buffering capacity. To test this, we employed a natural electrogenic Ca^2+^ ionophore ferutinin to promote mitochondrial Ca^2+^ overload independently of MCU and trigger Ca^2+^-dependent PTP opening^22,25^. PTP opening can be visualised by a significant drop in ΔΨm (Fig. 2a). Ferutinin was applied and the PTP in LRRK2 mutation bearing fibroblasts opened at significantly lower ferutinin concentrations. At 20 μM 62% of control cells ( ± 5.1%; *p* *<* 0.001; Fig. 2b) depolarised, whereas 94% (G2019S), 100% (R1441G) and 99% (Y1699C) were found to be depolarised. These results indicate that these LRRK2 mutations lead to a significantly lower Ca^2+^ buffering capacity when compared with control which may lead to an increased bioenergetic imbalance in cells with a high-energy demand such as neurons.

### Mitochondrial Ca^2+^ efflux deficiency not due to lower mitochondrial membrane potential

**Fig. 3:**
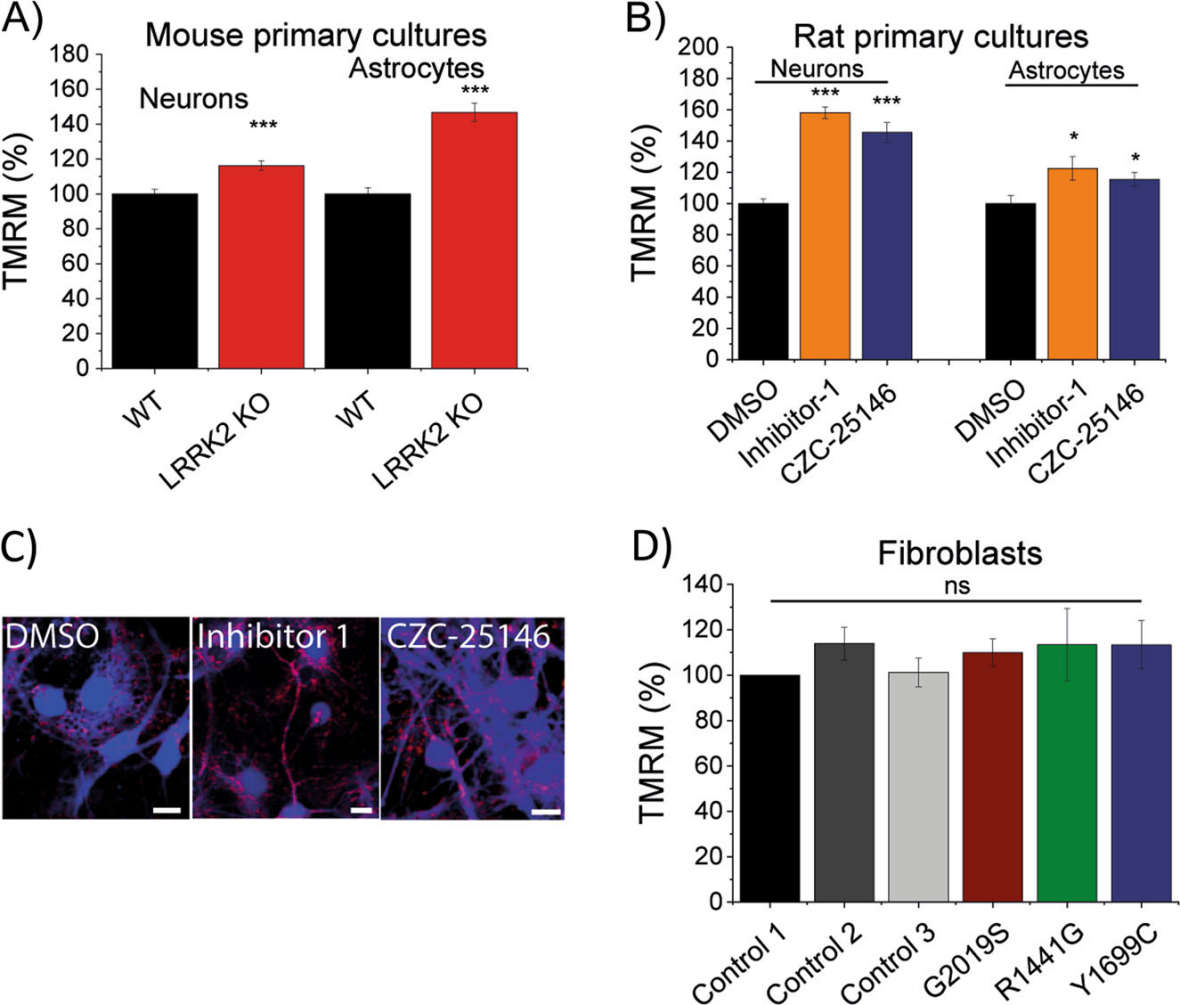
Ca^2+^ efflux deficiency not caused by lower MMP. **a** Quantification of MMP in WT and LRRK2 KO neurons (*N* = 3 experiments; *n* ≥ 60) and astrocytes (*N* = 3 experiments; *n* ≥ 41). **b** Quantification of MMP in rat WT neuronal co-cultures treated with either vehicle control DMSO, LRRK2-IN-1 or CZC-25146 (*N* = 3 experiments; *n* ≥ 45 cells). **c** Representative images of neurons and astrocytes treated with vehicle control DMSO, LRRK2-IN-1 or CZC-25146 and labelled with calcein blue and TMRM. **d** Quantification of MMP in control and LRRK2 mutation bearing fibroblasts (*N* = 3 experiments; *n* ≥ 42 cells). ***p *<* 0.001

Mitochondrial Ca^2+^ homoeostasis is tightly linked to mitochondrial membrane potential (ΔΨm) where an altered potential could lead to the observed inhibited mitochondrial Ca^2+^ efflux in LRRK2 deficiency. Therefore, we assessed ΔΨm in LRRK2 KO, LRRK2 inhibitor treated neuronal co-cultures and LRRK2 mutation bearing fibroblasts (Fig. 3). A significantly higher ΔΨm was recorded in LRRK2 KO neurons (116 ± 2.8%; *p* *<* 0.001) and astrocytes (146 ± 5.1%; *p* *<* 0.001) when compared with WT (Fig. 3a). Inhibition of LRRK2 with LRRK2-IN-1 and CZC-25146 resulted in a significantly increased ΔΨm in neurons (158 ± 3.6% and 145 ± 6.3%, respectively; *p* *<* 0.001) and astrocytes (122 ± 7.6% and 115 ± 4.5%, respectively; *p* < 0.05) when compared with cultures treated with DMSO vehicle control (Fig. 3b, c). Analysis of fibroblast bearing disease related LRRK2 mutations did not reveal a significant difference in ΔΨm to matched control fibroblasts (Fig. 3d).

### Upregulation of NCLX rescues the inhibited mitochondrial Ca^2+^ efflux

We have previously shown^16^ that direct and indirect modulation of NCLX activity can rescue a deficient mitochondrial Ca^2+^ efflux. Therefore, this study tested whether activation of NCLX is able to overcome the mitochondrial Ca^2+^ efflux phenotype observed in LRRK2 deficient models.

**Fig. 4:**
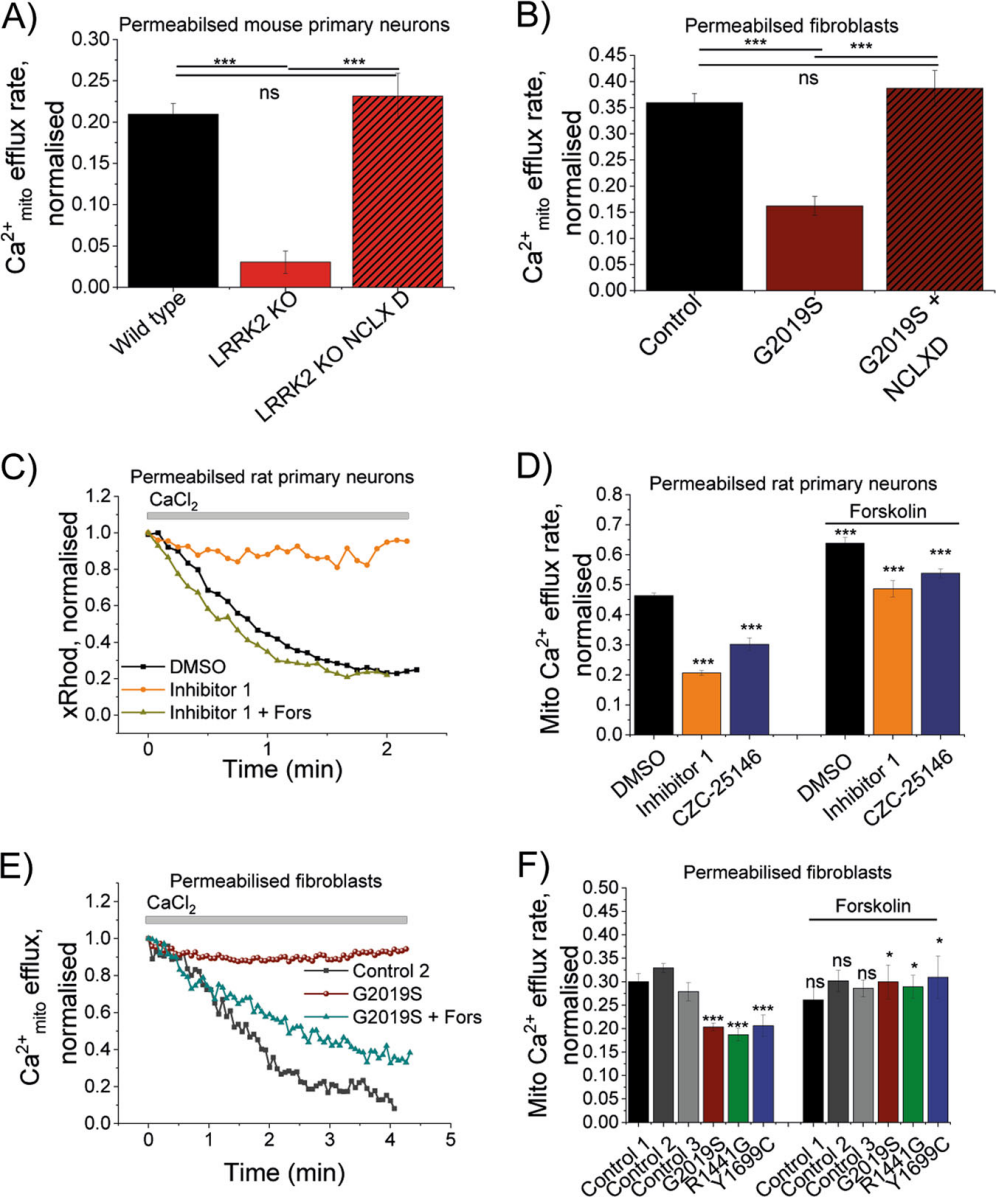
Direct and indirect upregulation of NCLX rescues mitochondrial Ca^2+^ efflux in all LRRK2 deficient models **a** Quantification of mitochondrial Ca^2+^ efflux rate in LRRK2 WT and KO primary neurons with or without NCLXD (*N* = 3 experiment; *n* ≥ 40 neurons). **b** Quantification of mitochondrial Ca^2+^ efflux rate in control or G2019S mutation bearing fibroblasts with or without NCLXD (*N* = 3 experiments; *n* ≥ 31 cells). **c** Representative traces of mitochondrial Ca^2+^ efflux in neurons treated with DMSO, LRRK2-IN-1 or CZC-25146 + pre-incubation with forskolin. **d** Quantification of mitochondrial Ca^2+^ efflux in neurons treated with DMSO, LRRK2-IN-1 or CZC-25146 + pre-incubation with forskolin (*N* = 3 experiments; *n* ≥ 59 cells). **e** Representative traces of mitochondrial Ca^2+^ efflux in permeabilised control, G2019S and G2019S + forskolin fibroblasts. **f** Quantification of mitochondrial Ca^2+^ efflux in permeabilised controls, G2019S, R1441G or Y1699C fibroblasts+/− forskolin (*N* = 4 experiments; *n* ≥ 59 cells). *p *<* 0.05; **p *<* 0.01 ***p *<* 0.001

Direct upregulation of NCLX is achieved by expression of a constitutively active NCLX. We have previously reported that the phosphomimetic mutant of NCLX (NCLX^S258D^) prevents mitochondrial Ca^2+^ overload in a PINK1 deficient model^16^. Hence, this study expressed NCLX^S258D^ in LRRK2 KO primary cultures and mitochondrial Ca^2+^ was recorded in neurons. Expression of NCLX^S258D^ restored the efflux back to levels similar to those observed in WT neurons (0.23 ± 0.02 and 0.2 ± 0.01, respectively; *p* *<* 0.001; Fig. 4a). Furthermore, expression of NCLX^S258D^ in patient fibroblast bearing the G2019S fibroblasts restored mitochondrial Ca^2+^ efflux back to unaffected control levels (0.38 ± 0.02 and 0.36 ± 0.01, respectively; *p* *<* 0.001; Fig. 4b).

An indirect upregulation of NCLX can be achieved through pre-exposure to the PKA activator forskolin. Forskolin activates adenylyl cyclase which in turn raises cyclic AMP (cAMP) levels. cAMP is required for PKA activation which in turn enables NCLX phosphorylation and therefore NCLX activation. WT neuronal co-cultures were pre-exposed to both LRRK2 inhibitors and forskolin before mitochondrial Ca^2+^ efflux was assessed in neurons (Fig. 4d). As previously observed, LRRK2-IN-1 (0.2 ± 0.01; *p* *<* 0.001) and CZC-25146 (0.3 ± 0.2; *p* *<* 0.001) inhibits mitochondrial Ca^2+^ efflux when compared with DMSO vehicle control (0.46 ± 0.01; *p* *<* 0.001; Fig. 4e). PKA-dependent upregulation of NCLX by application of forskolin restored the mitochondrial Ca^2+^ efflux deficiency in neurons treated with either LRRK2-IN-1 (0.48 ± 0.03; *p* *<* 0.001) or CZC-25146 (0.54 ± 0.01; *p* *<* 0.001).

Further, patient fibroblasts bearing the LRRK2 mutations were pre-exposed to forskolin and mitochondrial Ca^2+^ efflux was assessed. Forskolin restored the efflux rate in G2019S (0.29 ± 0.03), R1441G (0.29 ± 0.02) and Y1669C (0.31 ± 0.04) back to levels observed in unaffected control cell line (0.3 ± 0.017; Fig. 4e, f).

Forskolin was also able to restore the Ca^2+^ handling capacity in fibroblast bearing LRRK2 mutations upon ferutinin application (Fig. 2b). A significantly lower percentage of cells bearing LRRK2 mutations opened PTP when challenged with 20 μM ferutinin, whereas 66% of G2019S cells (versus 94% without forskolin; *p* *<* 0.001), 58% of Y1699C cells (versus 100% without forskolin; *p* *<* 0.001) and 60% of R1441G cells (versus 94% without forskolin; *p* *<* 0.01; *n* = 4 experiments) opened PTP at this forskolin concentration. Pre-incubation of control fibroblasts with forskolin also significantly increased the Ca^2+^ handling capacity in control 1 and control 3 cells (*p* *<* 0.05; Fig. 2b).

### NCLX modulation rescues Ca^2+^-dependent cell death

**Fig. 5:**
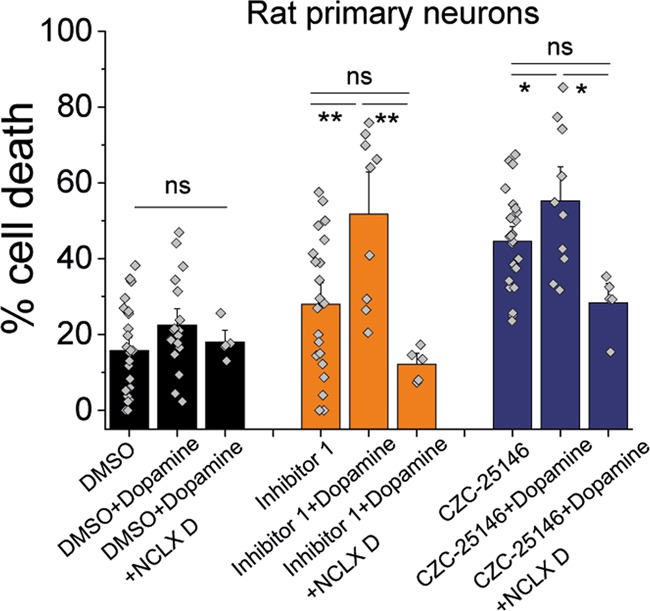
Protective effects of NCLX upregulation from dopamine-induced neuronal cell death. LRRK2 inhibitor treated neuronal cultures were challenged with 70 μM Dopamine for 12 h and cell death was assessed using PI and Hoechst (*N* = 3 experiments; each data point represents one field of view with an average of 58 neurons). *p < 0.05; **p < 0.01

Since inhibition of NCLX leads to a slower mitochondrial Ca^2+^ efflux we investigated whether the Ca^2+^ imbalance leads to neurotoxicity and ultimately cell death. We incubated primary neuronal co-cultures with DMSO, LRRK2-IN-1 or CZC-25146 and assessed neuronal cell death after 12 h. LRRK2-IN-1 increased neuronal cell death to 27.9% (± 3.8%) and CZC-25146 to 44.6% ( ± 2.6%) when compared with basal cell death of 15.7% ( ± 2.3%; Fig. 5). LRRK2 inhibition made neurons more prone to dopamine-induced cell toxicity and incubation of midbrain cultures with 70 μM dopamine triggered cell death, which was significantly higher in LRRK2 inhibited neurons (LRRK2-IN-1 51.8 ± 7.4%; CZC-25146 55.3 ± 5.9%) when compared with control (22.46 ± 2.9%). Since NCLX modulation rescued mitochondrial Ca^2+^ efflux we investigated whether upregulation is able to rescue dopamine-induced cell death. Indeed, expression of NCLX^S258D^ restored cell death in LRRK2 inhibited neurons back to control cell death levels (LRRK2-IN-1 12.1 ± 1.9%; CZC-25146 28.3 ± 5.9%).

## Discussion

Mitochondrial dysfunction has been linked to both familial and sporadic forms of PD. Several PD risk genes, such as PINK-1, Parkin and DJ-1 have been associated with mitochondrial pathogenesis observed in PD with a key role in mitochondrial Ca^2+^ homoeostasis^26–28^. Mitochondrial Ca^2+^ overload has a direct effect on mitochondrial bioenergetics and lowers the threshold for PTP opening leading to premature cell death^29,30^ and has previously been demonstrated in loss-of-function, recessively inherited mutations of PINK1^17,31^.

In this present study, we show that modulation of LRRK2 through deletion (KO), inhibition (de-phosphorylation) or mutagenesis of LRRK2 alters the mitochondrial Ca^2+^ efflux; therefore linking another PD risk gene to NCLX leading to decreased PTP opening threshold and an increased dopamine-induced neuronal death (Figs. 1, 2, 5). LRRK2 has previously been associated with mitochondrial membranes but no direct interaction between LRRK2 and NCLX could be confirmed in our previous manuscript (supplementary data) and is therefore unlikely to directly modulate NCLX Ca^2+^ efflux^11,16,32^.

We and other have previously described that a lower ΔΨ_m_ can result in reduced NCLX Ca^2+^ efflux^16,17^. However, ΔΨ_m_ in all LRRK2 models employed in this current study is either not changed (fibroblasts) or elevated (inhibitor/KO) when compared with control conditions suggesting that the ΔΨ_m_ is not the underlying reason for the mitochondrial Ca^2+^ efflux deficiency (Fig. 3). Papkovskaia et al.^33^ have previously reported that fibroblasts bearing G2019S bear a lower ΔΨ_m_. This current study employed three matched control cell lines as well as three mutations affecting different domains of LRRK2 (ROC, COR and kinase domain) and could not find any significant changes in the ΔΨ_m_.

A global Ca^2+^ dysregulation can lead to mitochondrial Ca^2+^ dysregulation, For example, Bedford et al.^34^ have provided evidence that LRRK2 stimulates voltage-gated Ca^2+^ channels, while the PD mutation G2019S leads to an increased channel stimulation when compared with wild-type LRRK2. Furthermore, LRRK2 has been proposed to stimulate plasma membrane sodium/calcium exchanger activity^35^ and nicotinic acid adenine dinucleotide phosphate receptors^36^. These reports provide evidence for a global Ca^2+^ regulation through LRRK2 where mutations can lead to a Ca^2+^ dysregulation affecting organelles and cellular functions. Indeed, Verma et al.^37^ has shown that mitochondrial Ca^2+^ contributes to dendrite injury in a LRRK2 PD model. The authors have also reported transcriptional upregulation the mitochondrial calcium uniporter and the mitochondrial calcium uptake 1 protein leading to increased mitochondrial Ca^2+^ influx. Interestingly, we did not observe an altered mitochondrial Ca^2+^ uptake in any of the models employed in this current study indicating other driving factor(s) for the mitochondrial Ca^2+^ efflux pathology (Fig. 1e, i, l).

The exact mechanism through which LRRK2 acts upon NCLX Ca^2+^ extrusion requires further investigation. LRRK2 has previously been reported to interact with the regulatory subunit of PKA, negatively regulating activity during synaptogenesis^38^. While LRRK2 is predominantly expressed in the cytosol, it has previously been suggested to affect mitochondrial dynamics^39,40^, with few studies providing evidence for a mitochondrial localisation^11,33^.

Our study has shown that mutations in LRRK2 phenocopy pharmacological inhibition and knockout of LRRK2 despite opposing reported effects (gain vs loss of function)^41,42^. One possible explanation may be link between LRRK2/Rab32 and PKA. It was shown that the small GTPase Rab32 interacts with the N-terminal armadillo repeat LRRK2. Rab32 itself has been reported to act as a PKA anchoring protein localising PKA to mitochondria and was shown to modulate mitochondrial associated membranes and mitochondrial fission^43,44^. How mutations or inhibition affect these interactions and the consequences on mitochondrial Ca^2+^ are yet to be unravelled. Therefore, the link between LRRK2/Rab32/PKA and its role in mitochondrial health in relation to PD requires further investigations.

We have previously shown that upregulation of NCLX via a cAMP/PKA-dependent activation through the application of forskolin rescues the mitochondrial Ca^2+^ efflux in PINK1 deficient models^16^. In this current study expression of the constitutively active phosphomimetic mutant of NCLX (NCLX^S258D^) also rescued the mitochondrial Ca^2+^ efflux in LRRK2 mutation bearing fibroblast and LRRK2 inhibitor treated cells in a similar manner previously described in a PINK PD model^16,45^ (Fig. 3). We and others have previously shown that cAMP-mediated PKA activation using forskolin reverses pathologies in a PINK1 and LRRK2 G2019S PD model^16,46^. This was mirrored in this current study as application of forskolin (indirect NCLX upregulation) restored the Ca^2+^ buffering capacity back to control levels in LRRK2 mutation bearing fibroblasts (Fig. 2). Delayed mitochondrial Ca^2+^ efflux, as described in this study, can lead to mitochondrial swelling and a lowered PTP opening threshold. PTP opening can be either triggered by high Ca^2+^ levels and or high superoxide levels and is characterised by the loss of the mitochondrial membrane potential, swelling of the mitochondrial matrix, rupture of the outer membrane and release of internalised Ca^2+^ and pro-apoptotic proteins including cytochrome C. Therefore, opening of the PTP is the first trigger for the induction of cell death pathways (apoptosis and necrosis) which can have particular detrimental effects in cells with a high-energy demand such as dopaminergic neurons which are predominantly affected in PD^45,47^. Here, we have shown that LRRK2 inhibitors make neurons more prone to dopamine-induced cell death (Ca^2+^-dependent). By upregulation of NCLX and therefore enhancing mitochondrial Ca^2+^ efflux, we were able to rescue this dopamine-induced neuronal cell death (Fig. 5).

Inhibition of NCLX in midbrain neurons in LRRK2 mutation is specifically important because it enhances the mitochondrial calcium level that increase probability to open permeability transition pore in mitochondria and trigger cell death in response to additional stresses which are typical for PD pathology such as oligomeric α-synuclein and dopamine^48,49^.

This study provides evidence that mitochondrial Ca^2+^ efflux deficiency is a common phenotype observed in two PD-associated risk genes (PINK1 and LRRK2). This phenotype can be rescued by an upregulation of NCLX via a direct or indirect cAMP/PKA-dependent approach which may offer a novel therapeutic strategy in PD. The observed phenotype associated with LRRK2 modulation may be of particular importance when employing LRRK2 inhibitors as mitochondrial bioenergetics are likely to be negatively affected. Prolonged mitochondrial Ca^2+^ dysregulation has detrimental bioenergetic consequences and is of particular importance to high-energy-demanding cells such as neurons. Since mitochondrial Ca^2+^ dysregulation is a common phenotype in many neurodegenerative disease models NCLX and its regulators may serve as a novel therapeutic strategy.
